# Oral melatonin as a new tool for neuroprotection in preterm newborns: study protocol for a randomized controlled trial

**DOI:** 10.1186/s13063-021-05034-w

**Published:** 2021-01-22

**Authors:** Francesca Garofoli, Stefania Longo, Camilla Pisoni, Patrizia Accorsi, Micol Angelini, Salvatore Aversa, Camilla Caporali, Sara Cociglio, Annalisa De Silvestri, Elisa Fazzi, Vittoria Rizzo, Chryssoula Tzialla, Marco Zecca, Simona Orcesi

**Affiliations:** 1 Neonatal Unit and Neonatal Intensive Care Unit, Fondazione IRCCS Policlinico San Matteo, Piazzale Golgi 1, 27100 Pavia, Italy; 2 Child and Adolescence Neuropsychiatry Unit, Children’s Hospital, ASST Spedali Civili of Brescia, 25123 Brescia, Italy; 3 Neonatal Unit and Neonatal Intensive Care Unit, Children’s Hospital, ASST Spedali Civili of Brescia, 25123 Brescia, Italy; 4 Child Neurology and Psychiatry Unit, Department of Brain and Behavioral Sciences, University of Pavia, 27100 Pavia, Italy; 5 Child Neurology and Psychiatry Unit, IRCCS Mondino Foundation, 27100 Pavia, Italy; 6 Unit of Clinical Epidemiology & Biometry, Fondazione IRCCS Policlinico San Matteo, 27100 Pavia, Italy; 7 Department of Clinical and Experimental Sciences, University of Brescia, 25123 Brescia, Italy; 8 Clinical Chemistry Laboratory and Department of Molecular Medicine, Fondazione IRCCS Policlinico San Matteo, 27100 Pavia, Italy

**Keywords:** Preterm newborn, Infants, Melatonin, Malondialdehyde, Neuroprotection, Anti-oxidant, Neuro-cognitive development

## Abstract

**Background:**

Prevention of neurodevelopmental impairment due to preterm birth is a major health challenge. Despite advanced obstetric and neonatal care, to date there are few neuroprotective molecules available. Melatonin has been shown to have anti-oxidant/anti-inflammatory effects and to reduce brain damage, mainly after hypoxic ischemic encephalopathy. The planned study will be the first aiming to evaluate the capacity of melatonin to mitigate brain impairment due to premature birth.

**Method:**

In our planned prospective, multicenter, double-blind, randomized vs placebo study, we will recruit, within 96 h of birth, 60 preterm newborns with a gestational age ≤ 29 weeks + 6 days; these infants will be randomly allocated to oral melatonin, 3 mg/kg/day, or placebo for 15 days. After the administration period, we will measure plasma levels of malondialdehyde, a lipid peroxidation product considered an early biological marker of melatonin treatment efficacy (primary outcome). At term-equivalent age, we will evaluate neurological status (through cerebral ultrasound, cerebral magnetic resonance imaging, vision and hearing evaluations, clinical neurological assessment, and screening for retinopathy of prematurity) as well as the incidence of bronchodysplasia and sepsis. We will also monitor neurodevelopmental outcome during the first 24 months of corrected age (using the modified Fagan Test of Infant Intelligence at 4–6 months and standardized neurological and developmental assessments at 24 months).

**Discussion:**

Preterm birth survivors often present long-term neurodevelopmental sequelae, such as motor, learning, social-behavioral, and communication problems. We aim to assess the role of melatonin as a neuroprotectant during the first weeks of extrauterine life, when preterm infants are unable to produce it spontaneously. This approach is based on the supposition that its anti-oxidant mechanism could be useful in preventing neurodevelopmental impairment. Considering the short- and long-term morbidities related to preterm birth, and the financial and social costs of the care of preterm infants, both at birth and over time, we suggest that melatonin administration could lead to considerable saving of resources. This would be the first study addressing the role of melatonin in very low birth weight preterm newborns, and it could provide a basis for further studies on melatonin as a neuroprotection strategy in this vulnerable population.

**Trial registration:**

ClinicalTrials.gov NCT04235673. Prospectively registered on 22 January 2020.

## Administrative information

**Table:** 

Title {1}	Oral melatonin as a new tool for neuroprotection in preterm newborns: study protocol for a randomized controlled trial
Trial registration {2a and 2b}.	ClinicalTrials.gov Identifier: NCT04235673. Prospectively registered on 22 January 2020.
Protocol version {3}	v.4 – 11/2019
Funding {4}	This study is supported by grants from the Italian Ministry of Health RC 2020 to IRCCS Mondino Foundation, Pavia, Italy, limited to English review and publication fees. There is no further funding body supporting the design of the study, the collection, analysis and interpretation of the data and the drafting of the manuscript.
Author details {5a}	^1^ Neonatal Unit and Neonatal Intensive Care Unit, Fondazione IRCCS Policlinico San Matteo, 27100 Pavia, Italy. ^2^ Child and Adolescence Neuropsychiatry Unit, Children’s Hospital ASST “Spedali Civili” of Brescia, 25123 Brescia, Italy. ^3^ Neonatal Unit and Neonatal Intensive Care Unit, Children’s Hospital, University Hospital ASST “Spedali Civili” of Brescia, 25123 Brescia, Italy. ^4^ Child Neurology and Psychiatry Unit, Department of Brain and Behavioural Sciences, University of Pavia, 27100, Pavia, Italy. ^5^ Child Neurology and Psychiatry Unit, IRCCS Mondino Foundation, 27100 Pavia, Italy. ^6^ Unit of Clinical Epidemiology & Biometry, Fondazione IRCCS Policlinico San Matteo, 27100, Pavia, Italy. ^7^ Department of Clinical and Experimental Sciences, University of Brescia, 25123, Brescia, Italy. ^8^ Clinical Chemistry Laboratory and Department of Molecular Medicine, Fondazione IRCCS Policlinico San Matteo, 27100 Pavia, Italy.
Name and contact information for the trial sponsor {5b}	No external sponsor.
Role of sponsor {5c}	Not applicable because this is a spontaneous, no-profit study.

## Introduction

### Background and rationale {6a}

The birth rate in Italy is approximately 552,000/year and 1% of these newborns are very low birth weight infants (≤ 1500 g at birth) or born at a gestational age (GA) of ≤ 29 weeks + 6 days [1]. Preterm birth survivors often present long-term neurodevelopmental sequelae, such as motor, learning, social-behavioral and communication problems [2–4]. Diffuse white matter (WM) damage, connectivity defects, and dysmaturational disturbances of cerebral WM and gray matter structures are now recognized to constitute the neuropathological substrate of these difficulties [5]. Magnetic resonance image (MRI) findings confirm that the WM is very susceptible to preterm birth-related injury [6], and recent studies [5] have shown that interventions aiming to prevent or mitigate “encephalopathy of prematurity” (i.e., this combination of WM injury and gray matter structural disturbances) may be possible.

Among the most promising molecules in this scenario, melatonin (ME) can be considered a prime candidate for conferring neuroprotection in preterm infants. This hormone and its metabolites are highly efficient anti-oxidants and free radical scavengers. ME is metabolized in the liver, but can also be metabolized by free radicals and oxidants and converted into c3-hydroxyME, which, in turn, directly scavenges free radicals. In the brain, it is metabolized into kynurenine derivatives, which are responsible for its anti-oxidant/anti-inflammatory response [7, 8]. ME has already been shown to act as a neuroprotectant in adult cerebral ischemia [9], and an increasing number of studies have consistently demonstrated that ME protects the developing brain by preventing the abnormal myelination and inflammatory glial reaction [10, 11] that are the main causes of WM injury [12]. ME has also been shown to be capable of reducing brain damage after neonatal hypoxic ischemic encephalopathy (HIE), and of preventing preeclampsia-related impairments, intrauterine fetal growth restriction, ventilation-induced damage and bronchopulmonary dysplasia (BPD) [13–16]. In prospective trials on newborns with HIE [17, 18], intravenous ME was shown to improve brain injury outcomes and significantly reduce concentrations both of the lipid peroxidation product malondialdehyde (MDA), and of nitrite/nitrate. Moreover, numerous ongoing trials dealing with ME administration in neonates (registered on ClinicalTrials.gov) report the absence of adverse events (AEs) linked to ME, and confirm its usefulness in the neonatal setting (i.e., in HIE). The fetus does not secrete ME but receives it by transplacental transfer, according to the maternal circadian secretion pattern. Functional production of ME is not established until about 12 weeks after birth, although a breastfed newborn can take advantage of maternal ME, whose concentration increases at night [19]. Conversely, preterm infants are deprived of the maternal ME they would normally have received during the last weeks/months of pregnancy and, moreover, their endogenous production of the hormone may be delayed even by months [20]. Therefore, exogenous ME administration during the first weeks of extrauterine life could help in preventing/mitigating the negative effects of prematurity on brain development.

Moreover, the pharmacokinetics of ME in preterm infants [19] show prolonged half-life and clearance and a decreased volume of distribution. The results of another pharmacokinetic study [21] highlighted the possibility of maintaining active concentrations of ME in blood through a single oral administration repeated every 12/24 h.

Until now, no study in very preterm infants has attempted to assess the capacity of ME to protect the brain against oxidative stress-induced impairment caused by preterm birth. This is the aim of our project, as we believe that improved knowledge in this area could influence, and even permanently modify, neurodevelopmental outcome in preterm infants. From the relevant literature on preterm [19, 21] and term infants [13–18], we merged all the available information about dosage, administration route, dosing interval and duration of treatment. Considering the novelty of the trial, the dosage of 3 mg/kg/ day for 15 days has been chosen.

### Objectives {7}

This study has 3 specific aims:

1. To measure, in a sample of preterm newborns, after 15 days of blind administration of ME or placebo, plasma MDA and its correlation with plasma ME (primary aim of the study).
2. To evaluate at term-equivalent age (TEA 40 weeks) whether, after 15 days of ME administration, infants have a better clinical and instrumental outcome, as shown by a reduction of neonatal morbidities such as BPD, infection/sepsis, hearing impairment, visual impairment (retinopathy of prematurity (ROP) or abnormal visual behavior), pathological neurological examination, and brain injury evaluated with cerebral ultrasound (cUS) and brain MRI.
3. To assess the infants’ neurodevelopmental outcome, correlating the results with the ME vs placebo treatment. More specifically, at 4–6 months of corrected age (CA), we will assess novelty preference and fixation duration using the eye-tracking-modified Fagan Test [22], which is moderately predictive of later cognitive abilities; at 18–24 months of CA, outcome will be assessed using anthropometric measurements, neurological and developmental evaluations and standardized developmental surveys administered to parents.

### Trial design {8}

The planned study will have a superiority, prospective, multicenter, parallel-groups, randomized, double-blind vs placebo-controlled design. Participants will be randomly allocated to the intervention group or the control group, in a 1:1 ratio. The intervention period will last 15 days. Preterm comorbidities and neurodevelopmental outcome will be evaluated throughout the first 24 months of CA. Table 1 shows the assessments to be administered at baseline and immediately post-intervention. This trial protocol is designed in accordance with the Standard Protocol Items: Recommendations for Interventional Trials (SPIRIT) 2013.

**Table 1:**
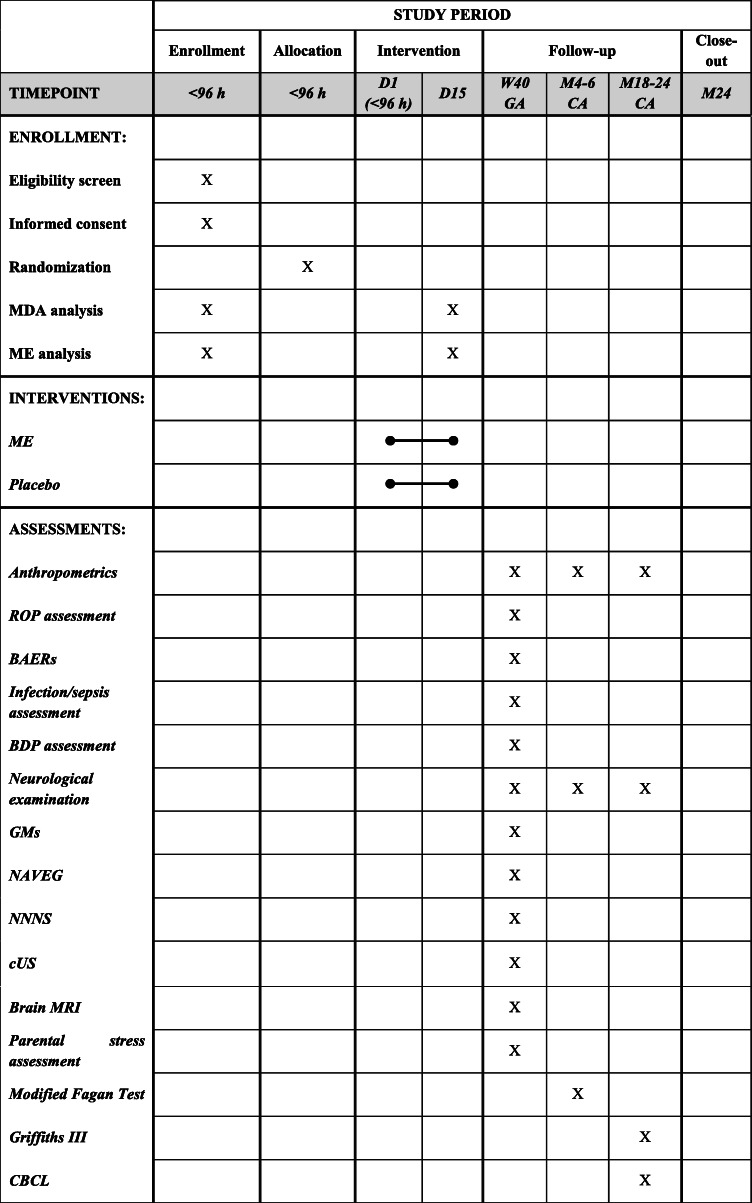
Standard Protocol Items: Recommendations for Interventional Trials (SPIRIT) figure with timeline for recruitment, assessments, and interventions

*Abbreviations*: *BAERs* brainstem auditory evoked responses, *BDP* bronchopulmonary dysplasia, *CA* corrected age, *CBCL* Child Behavior Checklist, *cUS* cerebral ultrasound, *D* day, *GA* gestational age, *GMs* general movements, *M* month, *MDA* malondialdehyde, *ME* melatonin, *MRI* magnetic resonance image, *NAVEG* Neonatal Assessment Visual European Grid, *NNNS* Neonatal Intensive Care Unit Network Neurobehavioral Scale, *ROP* retinopathy of prematurity, *W* week

## Methods: participants, interventions, and outcomes

### Study setting {9}

The role of coordinating center will be shared by the Fondazione IRCCS Policlinico San Matteo, Pavia, and the IRCCS Mondino Foundation, Pavia, while the collaborating center will be the ASST “Spedali Civili” Children’s Hospital in Brescia (where the local ethics committee approval process is still ongoing). Preterm newborns will be recruited within 96 h of birth at the two centers’ neonatal intensive care units (NICUs). ME and placebo will be administered at these NICUs. Plasma concentrations of MDA and ME will be determined at the laboratory of the coordinating center. Staff at the collaborating center’s NICU will therefore send cryopreserved neonatal plasma samples to the coordinating center. The assessments scheduled to take place at TEA will be performed at the NICU of birth. Follow-up assessments, scheduled to take place at 4–6 months and at 18–24 months of CA, will be performed at the Child Neuropsychiatry Units of Pavia and Brescia.

### Eligibility criteria {10}

Thirty preterm newborn infants will be enrolled in each of the two study arms. The enrolled infants will randomly receive ME (3 mg/kg/day) or placebo for 15 days.

The inclusion criteria are:

- GA ≤ 29 + 6 (weeks + days)
- Ability to receive 20 ml/kg/day maternal or formula milk, within 96 h of birth
- Written informed consent signed by both the parents.

The exclusion criteria are:

- GA > 29 + 6 (weeks + days)
- Inability to receive minimal enteral nutrition
- Genetic, congenital, metabolic, and/or TORCH diseases
- Periventricular/intraventricular hemorrhage (PVH/IVH) > grade III [23] and/or periventricular leukomalacia (PVL) ≥ grade II [24] within 96 h of birth (as assessed by cUS)
- Informed consent denied.

### Who will take informed consent? {26a}

NICU clinicians participating in the investigation will recruit participants and collect informed consent from both the parents. Parents will be fully informed about the study during a counseling session, and then given an information sheet.

### Additional consent provisions for collection and use of participant data and biological specimens {26b}

Not applicable as no ancillary studies are planned.

## Interventions

### Explanation for the choice of comparators {6b}

ME will be compared to placebo. Currently there are no other neuroprotectant molecules registered for use in NICU patients.

### Intervention description {11a}

The intervention will consist of double-blind administration of ME or placebo, according to a randomization list. Preterm infants eligible for inclusion after application of all the inclusion and exclusion criteria will be randomly assigned to receive 3 mg/kg/day of ME in drops or the equivalent amount of placebo in drops for 15 days. The study coordinator, after checking the list, will give the neonatologist the appropriate bottle of drops. Each bottle will be labeled “A” or “B” — in accordance with the double-blind administration protocol, neither staff nor patients (parents) will know which contain ME and which placebo, and will also show the patient’s identity, the date of the first administration, and the batch number. The neonatologist will establish and record the dose administered each evening on the basis of the patient’s weight, measured daily. A nurse will administer the drops at 11 p.m. each evening, after enteral feeding, for 15 consecutive days. This timing is intended to mimic maternal milk ME concentrations, which are highest during the night.

### Criteria for discontinuing or modifying allocated interventions {11b}

The study can be discontinued in a given trial participant due to the following: onset of AEs possibly related to the intervention, clinician judgment, or withdrawal of consent by parents.

### Strategies to improve adherence to interventions {11c}

ME/placebo will be administered for 15 days, during hospitalization in the NICUs, under the study investigators’ direct supervision and responsibility.

### Relevant concomitant care permitted or prohibited during the trial {11d}

All trial participants will receive the necessary standard of care: all treatments needed by patients, according to their clinical conditions, will be allowed. Every concomitant medication or intervention will be recorded by clinicians.

### Provisions for post-trial care {30}

A clinical and instrumental follow-up is planned until 24 months of CA, as reported in the “Intervention description” section and is part of the study. No ancillary or specific post-trial care is planned.

### Outcomes {12}

1. Outcomes relevant to the primary aim:
  A statistically significant decrease in plasma MDA concentration (nmol/ml) in the ME-treated group after 15 days of ME/placebo administration, constituting a difference between the two groups.
  At the same timepoint, a statistically significant increase in plasma ME concentration (μg/mL), assumed to be responsible for the above MDA decrease, constituting a difference between the two groups.
2. Outcomes relevant to the secondary aim: incidence, by 40 weeks of CA, of:
  Cases of cerebral injury, revealed by cUS and brain MRI.
  Cases of ROP, auditory deficit, sepsis, BPD
  Cases with abnormal neurological examination [25], abnormal general movements (GMs) [26], abnormal visual behavior, as assessed using the Neonatal Assessment Visual European Grid (NAVEG) [27].
3. Outcomes relevant to the third aim:
  ➣ At 4–6 months of CA:
    Visual function score, on the modified Fagan test [22].
  ➣ At 18–24 months of CA:
    Anthropometric measurements
    Neurodevelopment and behavior, as assessed using the Griffiths Scales of Child Development 3rd Edition [28] and the Child Behavior Checklist (CBCL) [29].

### Participant timeline {13}

See Table 1.

### Sample size {14}

The literature contains only one paper sufficiently similar to our study [30]; this paper was taken as a reference source to select the primary outcome (MDA concentration in the two groups) and calculate the sample size for our proposed study. A total of 26 patients per group is deemed sufficient to detect, with a power greater than 80%, a statistically significant difference equal to 8 nmol/ml (standard deviation in the two groups equal to 7) between the null hypothesis (H_0_) of the same concentration of MDA 16 nmol/ml in the two groups and the alternative hypothesis (H_1_) of an MDA concentration reduction to 8 nmol/ml in group 2 (*t*-test for independent groups with alpha error = 5%). Considering a potential dropout rate of 15%, 30 newborns per group will be enrolled.

All the participating patients will be enrolled by the two participating NICUs.

### Recruitment {15}

The recruitment will be done at Fondazione IRCCS Policlinico S. Matteo, Pavia, Italy (joint coordinating center) and ASST "Spedali Civili” di Brescia, Brescia, Italy (participating center). The coordinating center obtained the necessary legal permission to start the trial earlier than the participating center, which is currently awaiting ethical approval. Both hospitals are third-level centers and accept newborns from 23 weeks of GA onwards. The principal investigator (PI) and the other neonatologists participating in the trial actively work on the wards, with schedule timing that guarantees the constant presence (24 h per day) of the PI or at least one of the co-investigators responsible for recruiting patients, giving adequate information to parents, and following the ongoing study. The centers have an incidence of about 4 to 8 eligible patients/month (patients with GA ≤ 29 weeks + 6 days, able to receive 20 ml/kg/day enteral feeding within 96 h of birth). This should guarantee recruitment of a total of 60 patients (even accounting for dropouts).

The two centers are similar in terms of available instruments, and both have a dedicated child neurology and psychiatry service. The researchers involved in the study have similar high levels of expertise, underwent the same specialist training, and use same methodological tools. Furthermore, most of them have often worked together on other research projects over the past years.

## Assignment of interventions: allocation

### Sequence generation {16a}

The randomization will be generated by the Unit of Clinical Epidemiology and Biometry (unblinded team) at the coordinating center, according to a 1:1 allocation ratio, by means of computer-generated random numbers that will allow for extra patient allocations in the event of dropouts. The randomization sequence will be generated by a random number generator at the same unit. The PIs of the two NICUs will sequentially allocate the participants to the two groups on the basis of the generated lists.

### Concealment mechanism {16b}

The Clinical Epidemiology and Biometry Unit staff generating the randomization list will serve as the unblinded team, responsible for generating and also storing the randomization list and for communicating with other co-investigators or with ME/placebo suppliers if necessary. ME/placebo will be kept in anonymous vials, bearing alpha-numeric codes, lot numbers, and expiration dates. These vials will be logged and stored in a locked room at each NICU.

### Implementation {16c}

The local study coordinator, after checking patient’s eligibility and randomization list, gives the appropriate bottle of drops to the neonatologist. Each bottle will be labeled “A” or “B” and will also indicate the patient’s identity, the date of the first administration, and the batch number.

## Assignment of interventions: blinding

### Who will be blinded {17a}

With the exception of the staff of the Unit of Clinical Epidemiology and Biometry at the coordinating center, the entire study staff (health care professionals, outcome assessors and data analysts) will be blinded, as will each participant’s parents. The Unit of Clinical Epidemiology and Biometry will be responsible for unblinding in case of necessity (severe AEs possibly related to intervention).

### Procedure for unblinding if needed {17b}

Unblinding will be performed on completion of the study. Under normal circumstances, all participants will be unblinded simultaneously once the database is complete. Urgent, unplanned unblinding prior to this point will be done if needed to ensure participant safety, e.g., to identify the drug involved during an acute reaction. Unblinded information will be shared only on a need-to-know basis.

## Data collection and management

### Plans for assessment and collection of outcomes {18a}

All the trial staff members are well trained in protocol procedures and requirements of Good Clinical Practice (GCP), Good Laboratory Practice, and Good Experimental Practice guidelines and have already worked on clinical trials; moreover, the trial will be monitored, throughout its course, by the coordinating center’s ethics committee quality assurance team (ECQT). All staff members involved in the study are part of a clinical team that routinely takes care of preterm infants from 23 weeks of GA. Every operator involved in outcome evaluation has consolidated experience in the use of the chosen tools and has undergone specific training, when required (e.g., for assessment of GMs or for performing the NAVEG protocol for assessment of visual abilities). All the tests, procedures, and clinical evaluations that will be performed are validated. In the following paragraph, all the assessments to be used for collection of the outcomes are detailed and supported by the relevant references, confirming their validation and current use.

#### Experimental design aim 1

In accordance with the study’s double-blind, parallel-arm design, a total of 60 preterm newborns with GA ≤ 29 + 6 (weeks+days), if able to receive 20 ml/kg/day enteral feeding within 96 h of birth, will be randomly assigned to receive 3 mg/kg/day ME drops, or the equivalent amount of placebo drops, for 15 days. At the end of the 15 days, we aim to assess differences in plasma concentrations of MDA and ME in the supplemented vs the not supplemented group and vs the baseline situation.

At recruitment and then after 15 days of treatment, 250 μl plasma samples will be obtained from 500 μl neonatal blood samples by means of ethylene-diamine-tetra-acetic acid. These plasma samples will be used to measure MDA concentration (nmol/ml), by high-performance liquid chromatography (HPLC, Chromsystems Instruments & Chemicals; Grafelfing/Germany), and ME concentration (pg/ml), quantified in one chromatographic run by HPLC with fluorimetric detection [31] (a LC-10AD pump, an RF-551 fluorescence detector, and a SIL-10AD autosampler, Shimadzu Italia, Italy).

All the measurements will be performed at the coordinating center’s Clinical Chemistry Laboratory (Fondazione IRCCS Policlinico San Matteo, Pavia, Italy).

#### Experimental design aim 2

Obstetric and perinatal data will be recorded for each infant according to the Vermont Oxford Network criteria [32]. Moreover, at TEA (40 weeks), clinical and instrumental outcomes will be evaluated by collecting/performing the following:

- Anthropometric measurements;
- Incidence and grade of ROP, measured by indirect ophthalmoscopy (Ophthalmic Imaging System, RetCam 3, Clarity Medical Systems, Pleasanton, CA, USA);
- Incidence of hearing abnormality, evaluated with brainstem auditory evoked responses (Otodynamics Audiology Systems, Hatfield, Herts, UK);
- Incidence and severity of infection/sepsis;
- Incidence and severity of BPD;
- Results of neurological examination, conducted according to the Amiel-Tison method [25];
- Assessment of GMs [26];
- Assessment of visual behavior, performed using a new standardized tool (NAVEG) and investigating different aspects of neonatal visual function [27];
- Administration of the Neonatal Intensive Care Unit Network Neurobehavioral Scale [33];
- cUS findings: PVH/IVH as classified by Papile [23], PVL as classified by de Vries [24], and ventricular dilatation according to Ment [34] (Ultrasound model: Hitachi ALOKA Arietta V70, Hitachi Medical Systems Europe Holding AG, Zug, Switzerland, www.hitachi-medical-systems.com). cUS findings will also be classified and grouped according to Rademaker [35];
- Brain MRI during spontaneous sleep, performed using a 1.5-Tesla MRI scanner (Siemens) by radiographers blinded to participant allocation to the placebo or ME group; qualitative and quantitative volumetric techniques will be used to estimate overall and regional brain volumes for different tissue types; diffusion tensor imaging will be used to evaluate the integrity and maturation of WM by apparent diffusion coefficient and fractional anisotropy. Harmonization of scans at the different centers will be guaranteed.
- Parental stress assessment with validated surveys, as a control factor, in order to take into account the influence of parental stress on the children’s development [33]. This will be done by administering the Parental Stressor Scale: Neonatal Intensive Care Unit, the Modified Perinatal Post-traumatic Stress Disorder Questionnaire, the State Anxiety Inventory, and the Edinburgh Postnatal Depression Scale. All the surveys are validated and will be administered by trained personnel.

#### Experimental design aim 3

At 4–6 months of CA, we will collect/perform the following:

- Anthropometric measurements;
- Neurological examination;
- The modified Fagan Test of Infant Intelligence [22], which is moderately predictive of later cognitive abilities. The test will be carried out using an innovative new method involving automated eye tracking (Tobii TX 300, Tobii AB, publ, Danderyd, Stockholm, SE. https://www.tobiipro.com), aimed at ensuring accuracy and objectivity. The first target is novelty preference, which is the proportion of time the infant spends looking at a novel stimulus compared with the time spent looking at a previously presented stimulus. The second target is fixation duration, i.e., the mean duration of gaze at a stimulus.

At 18–24 months of CA, neurodevelopmental outcomes in the two groups will be evaluated through:

- Anthropometric measurements;
- Neurological examination;
- Standardized developmental assessment, performed using the Griffiths Scales of Child Development 3rd Edition [28];
- The CBCL, a self-administered survey completed by parents [29].

### Plans to promote participant retention and complete follow-up {18b}

The trial staff will be responsible for follow-up of patients. At the first follow-up (40 weeks of GA), the patients will mainly still be hospitalized. Subsequent follow-up visits will be planned with parents. In order to encourage compliance with the scheduled assessments, the study coordinator will telephone parents a week before each scheduled assessment. Parents will also be telephoned, with a reminder, should patients miss an appointment.

### Data management {19} and confidentiality {27}

All study-related information will first be collected in case report forms (CRFs) and then entered into the database by dedicated members of the trial staff. All collected data will then be stored securely at the coordinating center. Double-data entry by dedicated personnel will ensure correspondence between CRF and database data. The data value ranges used in the study will be those for preterm infants, checked by a neonatologist and by the data manager at the coordinating center. All participant information will be stored in locked filing cabinets in secure rooms accessible only to designated study staff (PIs, co-investigators, data analysts). All records that contain names or other personal identifiers will be stored separately from study records, anonymized and protected by an identification code which may be accessed only by authorized personnel. The participants’ study information will not be released outside the context of the present study without the written permission from the participant (parents).

### Plans for collection, laboratory evaluation, and storage of biological specimens for genetic or molecular analysis in this trial/future use {33}

Not applicable as no biological specimens for genetic or molecular analysis will be collected as part of this trial.

## Statistical methods

### Statistical methods for primary and secondary outcomes {20a}

With regard to analysis of the trial results, a normality test (Shapiro-Wilk test) will be used to determine whether datasets are well modeled by a normal distribution. Categorical variables will be described as counts and percentages and compared between treatment groups by chi-squared test or Fisher’s exact test. Continuous variables will be expressed as mean and standard deviation or median and interquartile range, and comparisons between treatment groups will be made using the *t*-test for independent groups or an analogous non-parametric test if the variable is not normally distributed. Potential multivariate analysis will be performed with regression models. To account for non-independence of observations in case twins are enrolled, general estimating equations (GEE) or mixed regression models will be used. Statistical analysis will be performed by the Clinical Epidemiology and Biometry Unit at the coordinating center using the STATA statistical package (Stata, release 16 or later, StataCorp, College Station, TX, USA).

### Interim analyses {21b}

No interim analyses have been planned as no problems likely to be detrimental to the participants are anticipated.

### Methods for additional analyses (e.g., subgroup analyses) {20b}

This is not applicable. There are no additional analyses planned.

### Methods in analysis to handle protocol non-adherence and any statistical methods to handle missing data {20c}

All randomized participants, regardless of protocol adherence, will be analyzed. Missing data will be handled with multiple imputation.

### Plans to give access to the full protocol, participant level-data, and statistical code {31c}

No later than 3 years after the end of the study (follow-up and statistical analysis phases included), we will deliver a completely de-identified dataset to an appropriate data archive for sharing purposes.

## Oversight and monitoring

### Composition of the coordinating center and trial steering committee {5d}

The coordinating center staff comprises neonatologists and child neuropsychiatrists responsible for patient enrollment and the assessment of clinical outcomes, and laboratory staff, researchers, and data analysts responsible for storing and providing placebo and ME to neonatologists, measuring biomarkers, collecting data, and compiling CRFs and the database. Together, all these staff members will coordinate the activity of the other physicians and researchers from the participating center.

The steering committee (SC) is composed of the PIs from each center and the “Support to Research Staff”. The SC’s duties are to oversee the advancement of the study, identify problems, and implement appropriate corrective measures.

### Composition of the data monitoring committee, its role and reporting structure {21a}

In our hospitals, we have dedicated data monitoring personnel who support the institutional ethics committees and PIs and Co-PIs; we refer, in particular, to the ECQT at the coordinating center. The said ECQT is external to the planned study and will actively monitor and audit the same through to the end of the follow-up. The personnel of the data monitoring committee (DMC) will monitor the ongoing study at regular intervals (twice a year).

Moreover, we declare that we have no sponsor. The planned trial is a spontaneous study and we have no competing interests.

### Adverse event reporting and harms {22}

All patients will receive treatment during NICU hospitalization and they will be under constant medical supervision. Some AEs are expected because of the nature of the study population: necrotizing enterocolitis, BPD, ROP, sepsis, death. No adverse reactions are expected from ME administration. Differences in the rates of the above AEs in the two study groups will be taken as an indicator of ME efficacy; these will be appropriately registered and entered in the statistical analysis as result (incidence of the events) and as AEs. All AEs will be managed in compliance with International Conference on Harmonization and GCP guidelines. The whole staff has been trained in the procedures and timing of registration of different types of AEs. Serious adverse reactions and suspected unexpected serious adverse reactions will be recorded and reported to the appropriate competent authorities, ethics committees and DMC within 24 h of their recognition.

All minor AEs will be reported and described in the CRFs and database, statistically analyzed and included in the results.

### Frequency and plans for auditing trial conduct {23}

The ECQT of the coordinating center will audit the study until the end of the follow-up.

### Plans for communicating important protocol amendments to relevant parties (e.g., trial participants, ethical committees) {25}

The staff of the coordinating center is responsible for communicating important protocol modifications to relevant parties. Any deviations from the protocol will be fully documented using a breach report form, and all updates of the protocol will be communicated between the coordinating and participating centers.

## Dissemination plans {31a}

We aim to publish quantitative and qualitative trial results in peer-reviewed scientific journals. Although no presentation of results at conferences/seminars is planned a priori, this possibility is not ruled out.

## Discussion

The combination of cerebral WM injury and dysmaturational events, both in WM and in neuroaxonal structures, resulting from preterm birth is a severe health concern. Preterm birth survivors often suffer long-term neurodevelopmental sequelae, such as motor, learning, social-behavioral and communication problems [2–4]. Families, too, are impacted by economic repercussions, which undoubtedly increase the lifelong psychological burden they have to bear. With regard to the entity known as “encephalopathy of prematurity”, it is evident that, in very preterm newborns in particular, the dysmaturational events evolve over a long period of time, which means that a relatively long window exists for interventions aimed at preventing or mitigating them [5]. Currently, however, there are few neuroprotective molecules available for treatment of these cases.

ME is a natural hormone with demonstrated anti-oxidant/anti-inflammatory properties, easy to administer (orally), inexpensive and with a high safety profile. As we have detailed above, different studies have demonstrated that ME is a candidate for WM injury prevention and, moreover, that preterm infants are deprived of maternal ME and also unable to secrete it. This extensive body of literature [7–18] leads us to strongly believe that exogenous ME administration could mitigate the effects of brain injury due to preterm birth, including long-term manifestations.

Considering the possible short- and long-term morbidities related to preterm birth and the financial and social costs of providing care for the preterm population at birth and over time, it can be hypothesized that ME administration, early after preterm birth, might result in considerable saving of social and economic resources.

Through our study, we aim to monitor the neurobehavioral development of infants over the first 2 years of life. We will focus on clinical signs and symptoms and on objective instrumental findings, comparing cases (ME-treated patients) with controls (placebo-treated patients). To establish whether ME supplementation can be considered a possible neuroprotective treatment against brain injury, we will try to determine which patients and disabilities benefited the most from ME supplementation and which the least, and to understand why. This study could be a step towards creating a preventive treatment tailored to the single patient’s characteristics, such as GA, birthweight, sex, or other variables and morbidities related to preterm birth.

It is underlined that the planned trial would be the first study addressing the role of ME in neuroprotection in preterm infants: we plan to evaluate different outcomes (metabolites, instrumental and clinical assessments), but some hypotheses may prove to be only partially demonstrable. Nevertheless, we are confident that our trial will produce results that may provide the basis for further studies on ME administration as a neuroprotection strategy. Another potential issue is that the incidence of preterm birth may follow an irregular sinusoidal curve, making it impossible to plan, with precision, the participant recruitment rate. In the event of a delay in recruiting the required number of participants, the time frame for enrollment may be extended from 12 to 18 months. Another solution could be to recruit patients from other centers that have NICUs and neuropsychiatry units, and use the same assessment methodology as we do.

In conclusion, this study aims to assess the possible neuroprotective role of ME in very preterm newborns. ME administration could potentially be an effective means of mitigating neurological impairments, strengthening the complex supportive therapy already provided in NICUs. Moreover, its preventive effects might also help to reduce national health system costs associated with the rehabilitation of developmental disabilities due to preterm birth.

## Trial status

The trial protocol refers to version v4–11/2019. Recruitment was expected to start in April 2020, but the current Coronavirus Disease-19 emergency delayed it. Sixty patients are expected to be recruited in the next 12–18 months and for each patient recruited, the follow-up will last 18–24 months, with 4 read-out points scheduled: at 15 days, at TEA, at 4–6 months of CA, and at 18–24 months of CA. Two extra months may possibly be needed for final analysis of data and reporting stages. We will deliver activity reports on each of the read-out points, and hold monthly follow-up meetings to track the progress.

The first milestone is 18 months after the start of the study: by this time, we aim to have completed the enrollment of the patients and accomplished Aims 1 and 2.

The second milestone is 36 months, by which time we expect to have accomplished Aim 3, collected the final results and completed the statistical analysis. As soon as the final results are available, the drafting of a scientific paper will begin.

## Abbreviations

BPD: Bronchopulmonary dysplasia
CA: Corrected age
CBCL: Child Behavior Checklist
CRFs: Case report forms
cUS: Cerebral ultrasound
DMC: Data monitoring committee
ECQT: Ethics committee quality assurance team
GA: Gestational age
GCP: Good Clinical Practice
GMs: General movements
HIE: Hypoxic ischemic encephalopathy
HPLC: High-performance liquid chromatography
IVH: Intraventricular hemorrhage
MDA: Malondialdehyde
ME: Melatonin
MRI: Magnetic resonance image
NAVEG: Neonatal Assessment Visual European Grid
NICUs: Neonatal intensive care units
PI: Principal investigator
PVH: Periventricular hemorrhage
PVL: Periventricular leukomalacia
ROP: Retinopathy of prematurity
SC: Steering committee
TEA: Term-equivalent age
WM: White matter
